# Hypoxia Increases Gefitinib-Resistant Lung Cancer Stem Cells through the Activation of Insulin-Like Growth Factor 1 Receptor

**DOI:** 10.1371/journal.pone.0086459

**Published:** 2014-01-28

**Authors:** Akiko Murakami, Fumiyuki Takahashi, Fariz Nurwidya, Isao Kobayashi, Kunihiko Minakata, Muneaki Hashimoto, Takeshi Nara, Motoyasu Kato, Ken Tajima, Naoko Shimada, Shin-ichiro Iwakami, Mariko Moriyama, Hiroyuki Moriyama, Fumiaki Koizumi, Kazuhisa Takahashi

**Affiliations:** 1 Department of Respiratory Medicine, Juntendo University, Graduate School of Medicine, Tokyo, Japan; 2 Research Institute for Diseases of Old Ages, Juntendo University, Graduate School of Medicine, Tokyo, Japan; 3 Department of Molecular and Cellular Parasitology, Juntendo University, Graduate School of Medicine, Tokyo, Japan; 4 Juntendo University Shizuoka Hospital, Shizuoka, Japan; 5 Pharmaceutical Research and technology institute, Kinki University, School of Medicine, Osaka, Japan; 6 Shien-Lab, National Cancer Center Hospital, Tokyo, Japan; Texas Tech University Health Sciences Center, United States of America

## Abstract

Accumulating evidence indicates that a small population of cancer stem cells (CSCs) is involved in intrinsic resistance to cancer treatment. The hypoxic microenvironment is an important stem cell niche that promotes the persistence of CSCs in tumors. Our aim here was to elucidate the role of hypoxia and CSCs in the resistance to gefitinib in non-small cell lung cancer (NSCLC) with activating epidermal growth factor receptor (EGFR) mutation. NSCLC cell lines, PC9 and HCC827, which express the *EGFR* exon 19 deletion mutations, were exposed to high concentration of gefitinib under normoxic or hypoxic conditions. Seven days after gefitinib exposure, a small fraction of viable cells were detected, and these were referred to as “gefitinib-resistant persisters” (GRPs). CD133, Oct4, Sox2, Nanog, CXCR4, and ALDH1A1–all genes involved in stemness–were highly expressed in GRPs in PC9 and HCC827 cells, and PC9 GRPs exhibited a high potential for tumorigenicity *in vivo*. The expression of insulin-like growth factor 1 (IGF1) was also upregulated and IGF1 receptor (IGF1R) was activated on GRPs. Importantly, hypoxic exposure significantly increased sphere formation, reflecting the self-renewal capability, and the population of CD133- and Oct4-positive GRPs. Additionally, hypoxia upregulated IGF1 expression through hypoxia-inducible factor 1α (HIF1α), and markedly promoted the activation of IGF1R on GRPs. Knockdown of IGF1 expression significantly reduced phosphorylated IGF1R-expressing GRPs under hypoxic conditions. Finally, inhibition of HIF1α or IGF1R by specific inhibitors significantly decreased the population of CD133- and Oct4-positive GRPs, which were increased by hypoxia in PC9 and HCC827 cells. Collectively, these findings suggest that hypoxia increased the population of lung CSCs resistant to gefitinib in *EGFR* mutation-positive NSCLC by activating IGF1R. Targeting the IGF1R pathway may be a promising strategy for overcoming gefitinib resistance in *EGFR* mutation-positive NSCLC induced by lung CSCs and microenvironment factors such as tumor hypoxia.

## Introduction

The acquisition of resistance to anticancer drugs remains a key obstacle for improving the prognosis of cancer patients. Drug resistance can occur through a variety of mechanisms, including drug efflux from cancer cells, augmented drug metabolism, secondary mutations in the drug target, and engagement of alternative survival pathways [1]. These mechanisms of acquired resistance are generally caused by genetic alterations within tumor cells, which persist during cancer treatment. However, recent studies have also revealed non-mutational mechanisms of drug resistance, including the existence of small population of cancer stem cells (CSCs) [2]. CSCs, which are also known as tumor-initiating cells and stem-like cancer cells, express stem cell markers including CD133, ABCG2, Bmi-1, and Oct4, and can form floating spheres in serum-free medium, a property associated with stem cells [3]–[5]. Increasing evidence indicates that small populations of CSCs are intrinsically more refractory to a variety of anticancer drugs and are responsible for the resistance to cancer treatment, which often accompanies tumor relapse [6]. Thus, targeting CSCs may improve treatment outcomes and lead to development of novel therapeutics for cancer patients.

Stem cell “niches” are defined as particular locations or microenvironments that maintain the properties of stem cell self-renewal and multipotency [7], [8]. Solid tumors often contain regions with insufficient oxygen delivery, a condition known as hypoxia, and several recent reports have suggested that hypoxia promotes the persistence of CSCs in tumors [9]. This hypoxic niche is responsible for CSC maintenance and plays a role in promoting therapeutic resistance [10]. Thus, targeting the hypoxic microenvironment may be another promising strategy for effective control of CSCs [9].

Advanced non-small cell lung cancer (NSCLC) is the leading cause of cancer-related deaths worldwide [11]. Somatic mutations in the epidermal growth factor receptor (EGFR) gene, such as an in-frame deletion mutation in exon 19, are associated with favorable response to the EGFR tyrosine kinase inhibitors (EGFR-TKIs), gefitinib and erlotinib [12]. EGFR kinase domain mutations occur at a significantly higher frequency in tumors from East Asians patients compared with non-Asians [13]. However, in most reports, progression-free survival of patients did not exceed 12 months, and most patients developed acquired resistance [14]. In addition, 25–30% of patients are intrinsically resistant to EGFR-TKIs as their tumors are diagnosed as harboring activating mutations in *EGFR* [15], [16]. The main mechanisms of resistance identified to date include secondary mutations and an “oncogene kinase switch.” The *EGFR* T790M mutation accounts for 50% of cases, and *MET* amplification can be detected in 20% of patients with *EGFR*-mutant TKI-resistant NSCLC [17]. However, the mechanisms responsible for intrinsic resistance and other acquired resistance to EGFR-TKI, as well as the issue of whether CSCs and hypoxic niches contribute to EGFR-TKI resistance, are not fully understood.

IGF1R is a transmembrane receptor tyrosine kinase responsible for cellular proliferation and survival [18]. IGF1R is expressed in many types of cancer cells, including NSCLC [18], and enhanced activation of IGF1R is implicated in resistance to chemotherapy and targeted therapies such as EGFR-TKIs [19], [20]. Several recent reports have suggested that IGF1R plays a critical role in the survival of some CSCs [21]. Chemoresistant colorectal cancer cells were enriched for CSCs and increased sensitivity to IGF1R inhibition [21]. According to Sharma et al., the drug-tolerant subpopulation following lethal drug exposure appeared to have the CSC phenotype and expressed phosphorylated IGF1R in several tested cell line models, including the NSCLC cell line PC9. Cotreatment of PC9 cells with EGFR-TKI plus IGF1R inhibitor prevented emergence of the drug-tolerant CSC phenotype [2]. Collective findings suggest a potential role for IGF1R signaling in the drug tolerance of CSCs to EGFR-TKIs. However, correlation between hypoxia, CSCs, and IGF1R signaling in EGFR-TKI resistance has not been clarified.

In this study, we examined the role of hypoxia in the persistence of lung CSCs in gefitinib resistance in NSCLC with activating *EGFR* mutations. The NSCLC cell lines PC9 and HCC827, carrying an activating *EGFR* mutation, were exposed to a high concentration of gefitinib under normoxic or hypoxic conditions. We found that hypoxia increased gefitinib-resistant lung CSCs in *EGFR* mutation-positive NSCLCs by activating IGF1R. The biological significance of hypoxia for the persistence of gefitinib-resistant CSCs and enhanced activation of IGF1R were investigated.

## Materials and Methods

### Cell Culture and Reagents

The NSCLC cell lines PC9 and HCC827, which express *EGFR* exon 19 deletion mutations (ΔE746–A750), were used in this study. PC9 cells were established at the Tokyo Medical University (Tokyo, Japan), as described previously [22], and were kindly provided by Dr. Kazuto Nishio (Department of Genome Biology, School of Medicine, Kinki University, Osaka). HCC827 cells were obtained from the American Type Culture Collection (Manassas, VA, USA). Cell lines were verified to be mycoplasma-free. Cells were cultured in RPMI-1640 medium (Wako, Osaka, Japan) supplemented with 10% fetal bovine serum (FBS), penicillin, and streptomycin (100 U/mL and 100 µg/mL, respectively), and were grown in a humidified 5% CO_2_ atmosphere at 37°C in an incubator, in which the oxygen tension was held at either 21% (normoxia) or 1% (hypoxia). Gefitinib was purchased from JS Research Chemicals Trading (Wedel, Germany). YC-1 and AEW541 were purchased from Sigma-Aldrich (St. Louis, MO, USA) and Selleck Chemicals (Houston, TX, USA), respectively.

### Generation of Gefitinib-resistant Persisters (GRPs)

A total of 2×10^5^ cells were plated in ten 10-cm plates and allowed to adhere for 24 hr. Cells were then treated with gefitinib at a concentration of 1 µM, which is 30–50 times the established IC_50_ values, and incubated under normoxia (21% O_2_) or hypoxia (1% O_2_). Media was replaced with Fresh media containing gefitinib every 3 days. Viable cells that remained attached on the dish at day 7 under normoxic or hypoxic conditions were considered to be gefitinib-resistant persisters (GRPs) or hypoxic gefitinib-resistant persisters (hypoxic GRPs), respectively, and were collected for analysis. The genetic identity of GRPs with the parental cells was confirmed using the GenomeLab Human STR Primer set from Beckman Coulter (Fullerton, CA, USA), according to the manufacturer’s instructions.

### Quantitative Real-time PCR

Total RNAs were extracted from cell lines using the mirVana miRNA Isolation kit (Ambion, Austin, TX, USA). cDNA was generated from 1 or 2 µg of RNA using the First-Strand cDNA Synthesis kit (GE Healthcare, Little Chalfont, UK) according to the manufacturer’s protocol. Quantitative real-time PCR (qPCR) was performed using Fast SYBR Green Master Mix (Applied Biosystems, Foster City, CA, USA) as previously described [22]. The following program was run: holding at 95°C for 20 sec, amplification via 40 cycles (denaturation 95°C for 3 sec, annealing, and extension at 60°C for 30 sec), with concurrent melt-curve analysis. We evaluated eleven housekeeping genes (*ACTB, GAPDH, UBC, B2M, YWHAZ, SF3A1, 28S rRNA, EIF4A2, SDHA, TOP1,* and *ATP5B*) in experimental samples to identify the most stably expressed control genes using geNorm software (http://medgen.ugent.be/~jvdesomp/genorm/). Actin beta (*ACTB*) and succinate dehydrogenase complex, subunit A flavoprotein (*SDHA*) were identified as the most stable housekeeping genes in our cell models by qPCR analysis; therefore, *ACTB* or *SDHA* were used as the internal controls.

The primers that were specific for the genes were as follows:

CD133

Forward, 5′-GGCCCAGTACAACACTACCAA-3′

Reverse, 5′-CGCCTCCTAGCACTGAATTGATA-3′

Oct4

Forward, 5′-GAGTGAGAGGCAACCTGGAG-3′

Reverse, 5′-GCCGGTTACAGAACCACACT-3′

Sox2

Forward, 5′-TACAGCATGTCCTACTCGCAG-3′

Reverse, 5′-GAGGAAGAGGTAACCACAGGG-3′

Nanog

Forward, 5′-TTTGTGGGCCTGAAGAAAACT-3′

Reverse, 5′-AGGGCTGTCCTGAATAAGCAG-3′

CXCR4

Forward, 5′-ACGCCACCAACAGTCAGAG-3′

Reverse, 5′-AGTCGGGAATAGTCAGCAGGA-3′

ALDH1A1

Forward, 5′-GCACGCCAGACTTACCTGTC-3′

Reverse, 5′-CCTCCTCAGTTGCAGGATTAAAG-3′

IGF1

Forward, 5′-GCTCTTCAGTTCGTGTGTGGA-3′

Reverse, 5′-CGACTGCTGGAGCCATACC-3′

### Immunofluorescence

PC9 or HCC827 cells were grown on Lab-Tek chamber II slides (Nunc, Rochester, NY, USA) with or without 1 µM or 2 µM gefitinib and under normoxic or hypoxic conditions for 72 h, fixed with 8% paraformaldehyde for 20 min, and permeabilized with 0.1% Triton X-100 for 3 min. After blocking with 10% goat serum in phosphate-buffered saline (PBS) for 30 min at room temperature, cells were incubated at 4°C overnight with the following primary antibodies: CD133 (Miltenyi Biotec, Bergisch Gladbach, Germany), Oct4 (Santa Cruz Biotechnology, Santa Cruz, CA, USA), IGF1 (Abcam, Cambridge, UK), and pIGF1R (Sigma-Aldrich). Proteins were visualized by incubation with secondary antibody labeled with Alexa Fluor 488 goat anti-rabbit IgG or Alexa Fluor 594 goat anti-mouse IgG (Invitrogen, Carlsbad, CA, USA). Slides were mounted using Vectashield Mounting Medium with DAPI (Vector Laboratories, Burlingame, CA, USA). Images were obtained on an Axioplan 2 imaging (ZEISS, Oberkochen, Germany) with AxioVision software (ZEISS). Images used for comparisons of different cells and/or treatments were acquired with the same instrument settings and exposure times and were processed similarly. The number of CD133-, Oct4-, and phosphorylated IGF1R-positive cells were counted; the ratio of positive cells was calculated in five fields for each experiment.

### Sphere Forming Assay

Single-cell suspension cultures were prepared at densities of 2.5×10^3^ cells per well in serum-free medium DMEM/F12 (Gibco, Grand Island, NY, USA) supplemented with commercial hormone mix B27 (Gibco), EGF (20 ng/mL; Gibco), FGF (20 ng/mL; Invitrogen), and heparin (2 µg/mL), and seeded into 6-well ultra-low attachment plates (Corning, Corning, NY, USA). PC9 parental cells and GRPs were incubated under normoxic conditions, while PC9 hypoxic GRPs were incubated under hypoxic conditions. Culture medium was replaced every 3 days; the number and size of the spheres were recorded and immunofluorescence analyses were performed 7 days after the start of the culture period [23].

### RNA Interference

Small interfering RNAs (siRNAs) targeting IGF1 (Stealth Select RNAi siRNA) were custom synthesized by Invitrogen. A negative control was also purchased from Invitrogen. PC9 or HCC827 cells were transfected with 2 different specific siRNAs and 1 non-specific control using Lipofectamine RNAiMAX (Invitrogen) according to the manufacturer’s instructions. The cells were detached and diluted in complete growth medium without antibiotics and then plated in each of the wells. RNAi duplex and Lipofectamine RNAiMAX were mixed in Opti-MEM®I (Gibco) reduced serum medium and incubated for 15 min at room temperature. RNAi duplex-Lipofectamine™ RNAiMAX complexes were added to the wells containing cells. The cells were then incubated for 48 h at 37°C.

The sequences of the siRNA against IGF1 were as follows:

IGF1 #1∶5′-ACACCAUGUCCUCCUCGCAUCUCUU-3′

IGF1 #2∶5′-UGCUGCUUCCGGAGCUGUGAUCUAA-3′

### Colony Inhibition Assay

PC9 cells (2×10^5^) or HCC827 cells (4×10^5^) were plated in 10-cm plates and allowed to adhere for 24 h. Cells were then incubated with 1 µM or 2 µM gefitinib, and with or without 0.01 µM, 0.1 µM, or 1 µM AEW541 under hypoxic conditions for 18 days for PC9 cells or 11 days for HCC827 cells. After incubation, the numbers of colonies were counted.

### *In vivo* Tumorigenicity Study

NOD/Shi-scid/IL-2Rcnull (NOG) mice (7-week-old, female) were purchased from the Central Institute for Experimental Animals (Kanagawa, Japan). All mice were shipped to the Juntendo University and were maintained under pathogen-free conditions. The mice were housed in a room under controlled temperature (25°C), humidity, and lighting (12-h light/dark cycle). *Ad libitum* access to food and tap water was allowed throughout the study period. To evaluate the *in vivo* tumorigenic potential, 1×10 cells or 1×10^2^ cells of PC9 parental cells and normoxic and hypoxic PC9 GRPs were mixed with Matrigel (BD Biosciences, San Jose, CA) and injected into both flanks of NOG mice. Tumor formation was evaluated 22–50 days after injection.

### Ethics

All animal experiments were carried out in accordance with the Fundamental Guidelines for Proper Conduct of Animal Experiment and Related Activities in Academic Research Institutions under the jurisdiction of the Ministry of Education, Culture, Sports, Science and Technology (Notice No. 71, 2006) and were approved by the Committee for Animal Experimentation of Juntendo University with the Approval No. 240182.

### Statistical Analysis

Values were compared using a two-tailed Student’s *t*-test. To compare multiple groups, a one-way analysis of variance (ANOVA) test was applied. Statistical analysis of sphere size was performed using the Kruskal-Wallis test. Differences between the means were considered statistically significant when p<0.05.

## Results

### Isolation and Gene Expression Analysis of Gefitinib-resistant Persisters (GRPs) Derived from NSCLC Cell Lines Harboring an Activating *EGFR* Mutation

**Figure 1:**
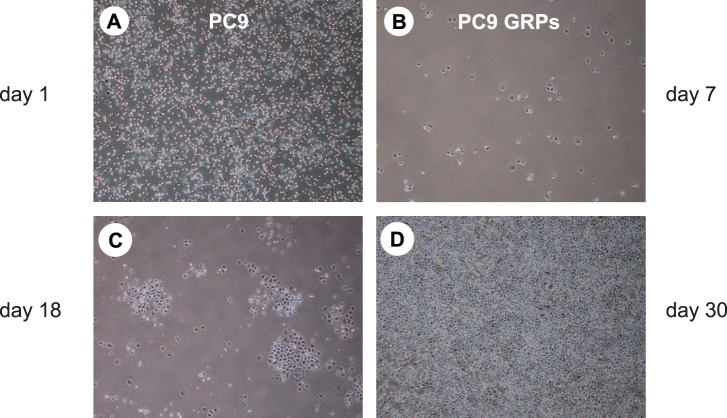
Isolation of gefitinib-resistant persisters (GRPs) derived from NSCLC cell lines harboring an activating *EGFR* mutation. First, 2×10^5^ PC9 cells were plated in 10-cm plates and allowed to adhere for 24 h (A). Cells were then treated with 1 µM gefitinib. Fresh media-containing gefitinib was replaced every 3 days. Seven days after gefitinib exposure, a small fraction of viable cells remained (B), resumed proliferating 18 days later (C), and continued to proliferate even 30 days later (D). **A.** Representative microscopic image of parental PC9 cells. **B, C, D.** Representative images of PC9 GRPs exposed to 1 µM gefitinib for 7, 18, and 30 days were photographed, respectively.

The NSCLC cell line PC9, carrying an activating *EGFR* mutation, was treated with 1 µM gefitinib. As described in a previous report [2], a small fraction of viable cells could survive and remain 7 days later, whereas most cells were dead within a few days (Figure 1B). We referred to these cells as gefitinib-resistant persisters (GRPs). Although GRPs were extremely quiescent, GRPs resumed proliferating under the high concentration of gefitinib (Figure 1C) and eventually showed the normal proliferation (Figure 1D). An analogous population of GRPs was detected in the other NSCLC cell line tested, HCC827 harboring a sensitive *EGFR* mutation, by treatment with 1 µM gefitinib (data not shown).

The genetic identity of GRPs with the parental cells were confirmed using a PCR-based analysis (GenomeLab Human STR Primer set from Beckman Coulter) that interrogates a set of 12 short tandem repeats. Analysis of genomic DNAs from parental cells and GRPs of PC9 and HCC827 cells are shown in Figure S1 in Information S1. STR profiles of the parental cells and GRPs are same. In addition, GRPs of PC9 and HCC827 cells retained the *EGFR* exon 19 deletion mutation (ΔE746–A750), as assessed by direct sequencing (data not shown). Taken together, these findings confirm that GRPs did not arise from contaminating cells. Neither the *EGFR* T790M mutation nor *MET* gene amplification was observed in GRPs in both PC9 and HCC827 cells (data not shown). The tyrosine kinase Src was not activated in GRPs of both PC9 and HCC827 cells compared with untreated parental cells (Figure S2 in Information S1). EGFR and HER3 were activated on parental PC9 and HCC827 cells, but expressions were suppressed markedly on the GRPs of both cell lines (Figure S2 in Information S1).

**Figure 2:**
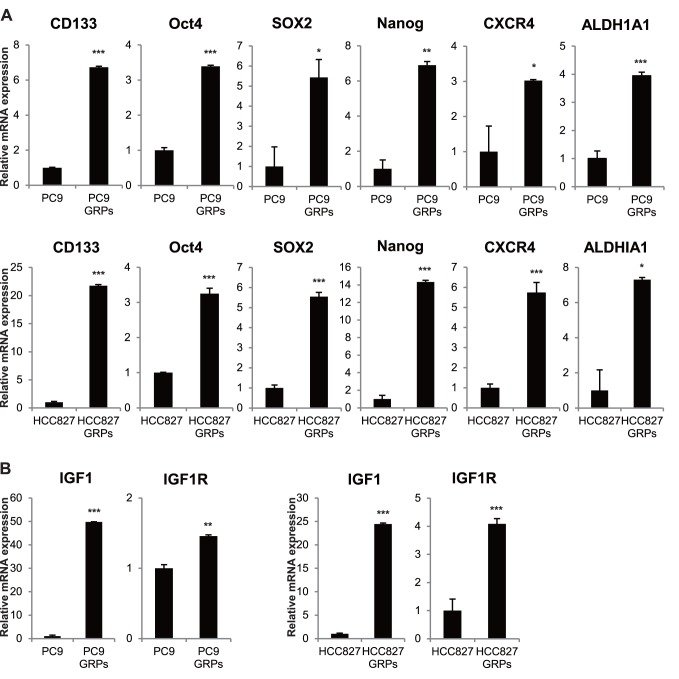
GRPs were highly enriched for gene expression of stemness, IGF1, and IGF1R. **A.** Quantitative RT-PCR was performed with primers specific for CD133, Oct4, Sox2, Nanog, CXCR4, and ALDH1A1, which are stemness genes in PC9 or HCC827 parental cells and GRPs. **B.** Quantitative RT-PCR was performed with primers specific for IGF1 and IGF1R in PC9 or HCC827 parental cells and GRPs. Data were normalized to actin expression. *p<0.01, **p<0.001, ***p<0.0001.

To identify the mechanism underlying gefitinib resistance in our cell model, we first analyzed gene expression in parental cells and GRPs using qPCR. The putative lung CSC marker CD133 was highly expressed in GRPs in PC9 and HCC827 cells (Figure 2A), suggesting a stemness phenotype. To further determine whether GRPs could reveal the characteristics of CSCs in our cell models, we evaluated the expression of genes regulating and maintaining the stem cell phenotype. Interestingly, gene expressions of Oct4, Sox2, and Nanog, which are also known to be involved in reprogramming mouse or human somatic cells to undifferentiated, pluripotent stem cells, were significantly increased in GRPs in PC9 cells, showing increases of 3.4-fold, 5.4-fold, and 6.9-fold, respectively, over parental PC9 cells (Figure 2A). Furthermore, expression of CXCR4 and ALDH1A1, also shown to be associated with the CSC phenotype, were upregulated (Figure 2A). Similar results were obtained for HCC827 GRPs (Figure 2A). Other known stem cell genes such as ABCG2, Bmi-1, and CD117 (c-Kit), were not upregulated in GRPs (data not shown).

A previous report also showed that IGF1R was phosphorylated and activated in EGFR-TKI-tolerant cells in PC9 cells and that an IGF1R inhibitor, AEW541, could prevent the emergence of these tolerant cells [2]. We examined IGF1 and IGF1R expression using qPCR in our cell model. IGF1 expression was dramatically upregulated in PC9 GRPs compared to PC9 parental cells, showing increase of 49.8-fold (Figure 2B). IGF1R expression was slightly increased in GRPs (Figure 2B). Similar results were obtained for GRPs in HCC827 cells (Figure 2B).

Taken together, these findings suggest that the small population of NSCLC cells that were highly enriched for gene expression of stemness and IGF1 survived and could resume proliferating under treatment a high concentration of gefitinib.

### Sphere-forming Ability and Tumorigenicity of GRPs were Upregulated and Sphere Formation by GRPs Increased after Exposure to Hypoxic Conditions

**Figure 3:**
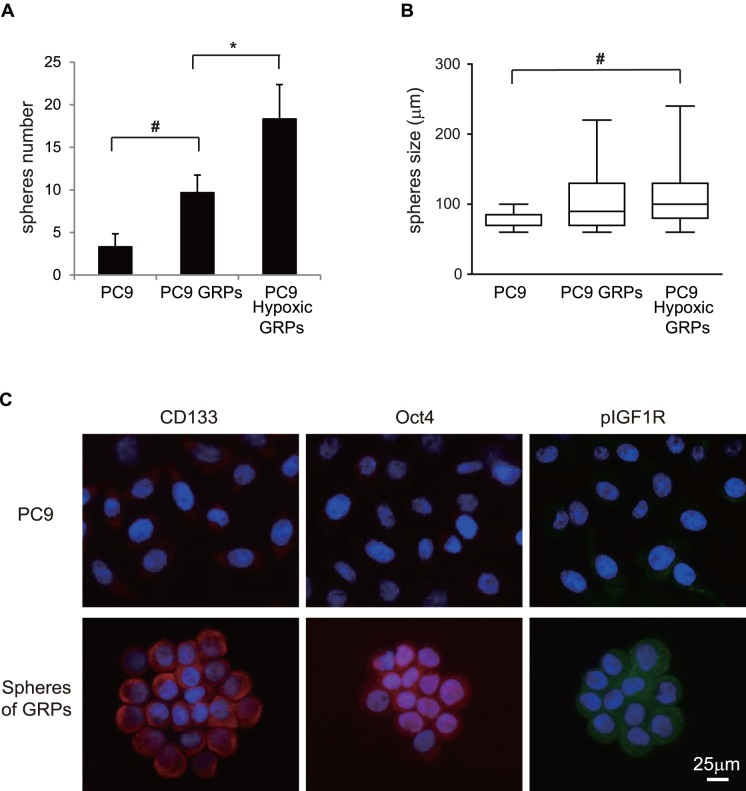
Sphere-forming ability of GRPs was upregulated and hypoxia increased this capacity. PC9 parental cells, GRPs, and hypoxic GRPs were prepared at densities of 2.5×10^3^ cells per 2 mL per well in serum-free media supplemented with growth factors and seeded into 6-well ultra-low attachment plates. PC9 parental cells and GRPs were incubated under normoxic conditions, while PC9 hypoxic GRPs were grown under hypoxic conditions. Culture medium was fed every 3 days. The number and size of spheres were recorded and immunofluorescence was performed 7 days after the start of the culture period. Spheres were fixed and incubated with primary antibodies against CD133, Oct4, or phosphorylated IGF1R (pIGF1R), and then with secondary antibody labeled with Alexa Fluor 594 goat anti-mouse IgG (red) or Alexa Fluor 488 goat anti-rabbit IgG (green). Cell nuclei were stained with DAPI (blue). Images were obtained on an Axioplan 2 imaging system with AxioVision software. **A.** The number of spheres was significantly increased in PC9 GRPs compared to in parental cells, and was further increased in PC9 hypoxic GRPs. *p<0.01, # p<0.05. **B.** Sphere size of PC9 hypoxic GRPs was significantly greater than that of parental cells. # p<0.05. **C.** Immunofluorescent images of control cells of PC9 (left) and spheres of GRPs (right) for CD133, Oct4, and pIGF1R.

To further evaluate the stemness of GRPs, sphere-forming assays reflecting the self-renewal activity and *in vivo* tumorigenicity studies were performed. The ability to form spheres was significantly higher in PC9 GRPs than in parental cells (Figure 3A). Importantly, hypoxia markedly increased the number of spheres of PC9 GRPs (Figure 3A). Sphere size of PC9 GRPs under hypoxic conditions was significantly larger than that of parental cells (Figure 3B). Immunofluorescence results showed that spheres of GRPs were positive for CD133, Oct4, and phosphorylated IGF1R (Figure 3C). In addition, we injected 1×10 cells or 1×10^2^ cells of parental PC9 cells and normoxic and hypoxic GRPs into both flanks of NOG mice and compared the tumorigenic potential *in vivo*. Tumor incidences of normoxic and hypoxic GRPs in mice were higher than those in parental cells (Table 1). Notably, normoxic and hypoxic GRPs formed tumors in two and five out of 16 NOG mice at 1×10 cells/injection, respectively, although the parental cells did not form tumors (Table 1). The difference in tumor formation between the parental cell group and hypoxic GRPs group with the injection of 1×10 cells was statistically significant (*p = 0.040). Taken together, these findings suggest that GRPs enriched for stemness markers have characteristic features of the stem cell phenotype and that the hypoxic microenvironment upregulated and maintained stem cell properties of GRPs, such as the sphere-forming ability and tumorigenicity.

**Table 1:** Tumor incidence of parental PC9 cells and normoxic and hypoxic GRPs transplanted into NOG mice.

	Tumor incidence/Number of injections	
Cells injected	1×10^1^	1×10^2^
Parental PC9 cells	0/16	6/16
PC9 GRPs	2/16	8/16
PC9 Hypoxic GRPs	5/16 #	9/16

GRPs; Gefitinib resistant persisters.

NOG mice; NOD/Shi-scid/IL-2Rcnull (NOG) mice.

# Tumor incidence was increased significantly in hypoxic GRPs group as compared with parental cell group with 1×10^1^ cells/injection, p<0.05.

To evaluate the *in vivo* tumorigenic potential, 1×10 cells or 1×10^2^ cells of parental PC9 cells or normoxic and hypoxic PC9 GRPs were mixed with Matrigel and injected into both flanks of NOG mice. Tumor formation was evaluated 33 days after injection.

### Population of CD133- and Oct4-positive GRPs were Increased Under Hypoxic Conditions

**Figure 4:**
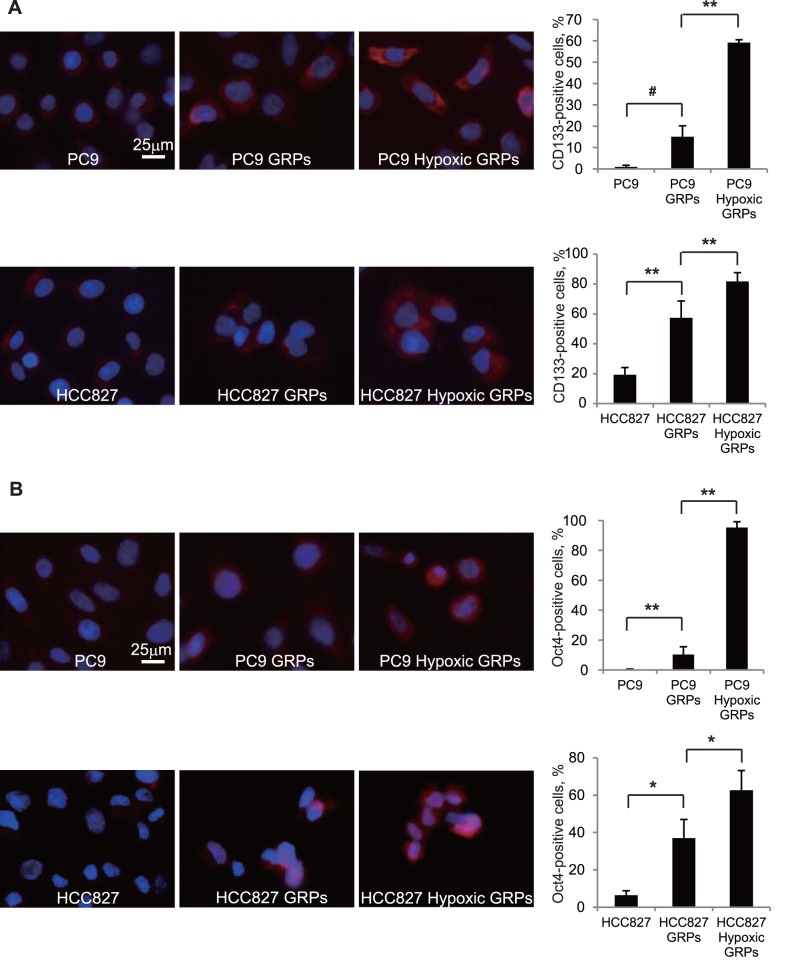
Population of CD133- and Oct4-positive GRPs were increased under hypoxic conditions. **A, B.** PC9 or HCC827 cells, growing on Lab-Tek chamber slides with or without 1 or 2 µM gefitinib and under normoxic or hypoxic conditions for 72 h, fixed, and incubated with the primary antibodies against CD133 (**A**) and Oct4 (**B**), and then with secondary antibody labeled with Alexa Fluor 594 goat anti-mouse IgG (red). Cell nuclei were stained with DAPI (blue). Images were obtained on an Axioplan 2 imaging system with AxioVision software. Images used to compare parental cells, GRPs, and hypoxic GRPs were acquired using the same instrument settings and exposure times and were processed similarly. The numbers of CD133 and Oct4-positive cells were counted, and the ratio of these cells was calculated in five fields in each experiment. **p<0.001, *p<0.01, # p<0.05.

To investigate the role of hypoxia on the generation and persistence of gefitinib-resistant stem cell population, we evaluated CD133- and Oct4-positive GRPs under normoxic and hypoxic conditions using immunofluorescence. As shown in Figure 4A and 4B, hypoxia significantly increased the population of CD133- and Oct4-positive GRPs in PC9 and HCC827 cells.

### IGF1R was Activated on GRPs Under Hypoxic Conditions, and Knockdown of IGF1 Decreased the Population of CD133- and Oct4-positive Hypoxic GRPs

**Figure 5:**
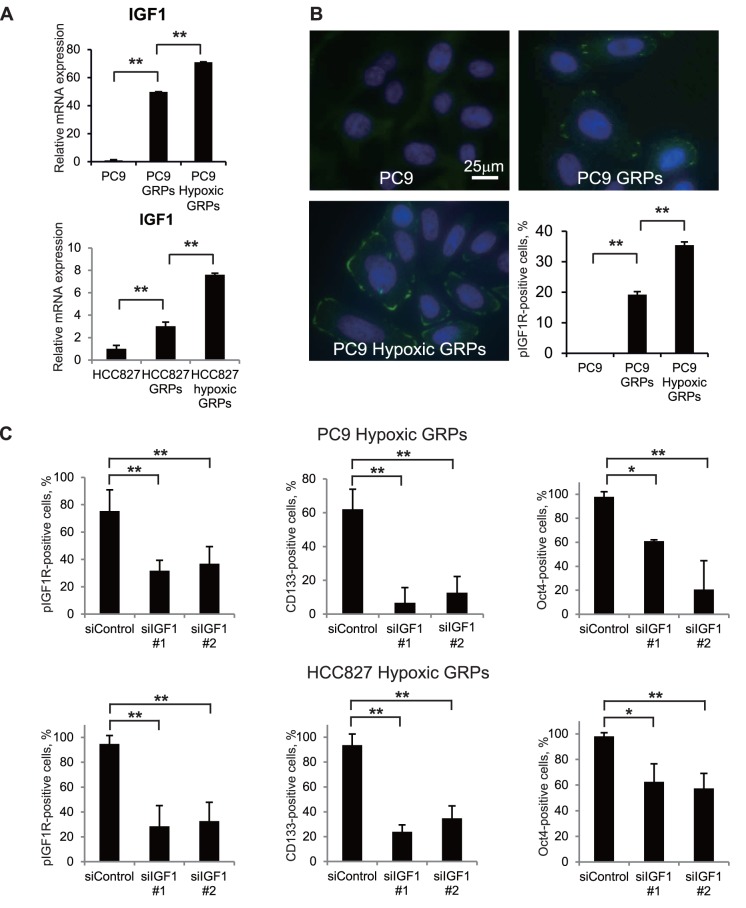
IGF1R was phosphorylated on hypoxic GRPs, and knockdown of IGF1 decreased the population of CD133- and Oct4-positive hypoxic GRPs. **A.** Quantitative RT-PCR was performed with primers specific for IGF1 in PC9 or HCC827 parental cells, GRPs, and hypoxic GRPs. Data were normalized to actin expression. **p<0.001. **B.** PC9 cells, grown on Lab-Tek chamber slides with or without 1 µM gefitinib and under normoxic or hypoxic conditions for 72 h, fixed, and incubated with the primary antibodies against phosphorylated IGF1R (pIGF1R) and then with secondary antibodies labeled with Alexa Fluor 488 goat anti-rabbit IgG (green). Cell nuclei were stained with DAPI (blue). Images were obtained using an Axioplan 2 system imaging with AxioVision software. Images used to compare PC9 parental cells, GRPs, and hypoxic GRPs were acquired with the same instrument settings and exposure times, and were processed similarly. The number of pIGF1R-positive cells was counted, and the ratio of these cells was calculated in five fields in each experiment. **p<0.001. **C.** IGF1 expression was knocked down in PC9 or HCC827 hypoxic GRPs by using small interfering RNA (siRNA) in Lab-Tek chamber slides. Immunofluorescence staining for pIGF1R, CD133, or Oct4 was then performed. Two specific siRNAs and one non-specific control were used. The numbers of pIGF1R-, CD133- and Oct4-positive cells were counted, and the ratio of these cells was calculated in five fields for each experiment. **p<0.001, *p<0.01.

To investigate the effect of hypoxia on the activation of IGF1R, we examined the gene expression of IGF1 in GRPs by qPCR as well as phosphorylation of IGF1R on GRPs using immunofluorescence under normoxic and hypoxic conditions. As shown in Figure 5A, IGF1 mRNA expression was much higher in PC9 GRPs than in parental PC9 cells and was upregulated by exposure to hypoxic conditions. Importantly, phosphorylated IGF1R was expressed on PC9 GRPs, and exposure to hypoxia markedly upregulated the population of phosphorylated IGF1R-expressing PC9 GRPs (Figure 5B).

To clarify the biological significance of IGF1 in gefitinib resistance in our hypoxic cell model, we knocked down IGF1 expression in hypoxic GRPs of PC9 and HCC827 cells using two different specific siRNAs (#1 and #2; Figure S3 in Information S1). As shown in Figure 5C, the knockdown of IGF1 significantly reduced phosphorylated IGF1R-expressing GRPs, and decreased CD133- and Oct4-positive GRPs under hypoxic conditions. These findings suggest that IGF1 plays a role in the activation of IGF1R and the maintenance of gefitinib-resistant CSCs.

### Hypoxia Regulates IGF1 Expression through HIF1α, and the Inhibition of HIF1α or IGF1R Decreased CD133- and Oct4-positive GRPs Under Hypoxia

**Figure 6:**
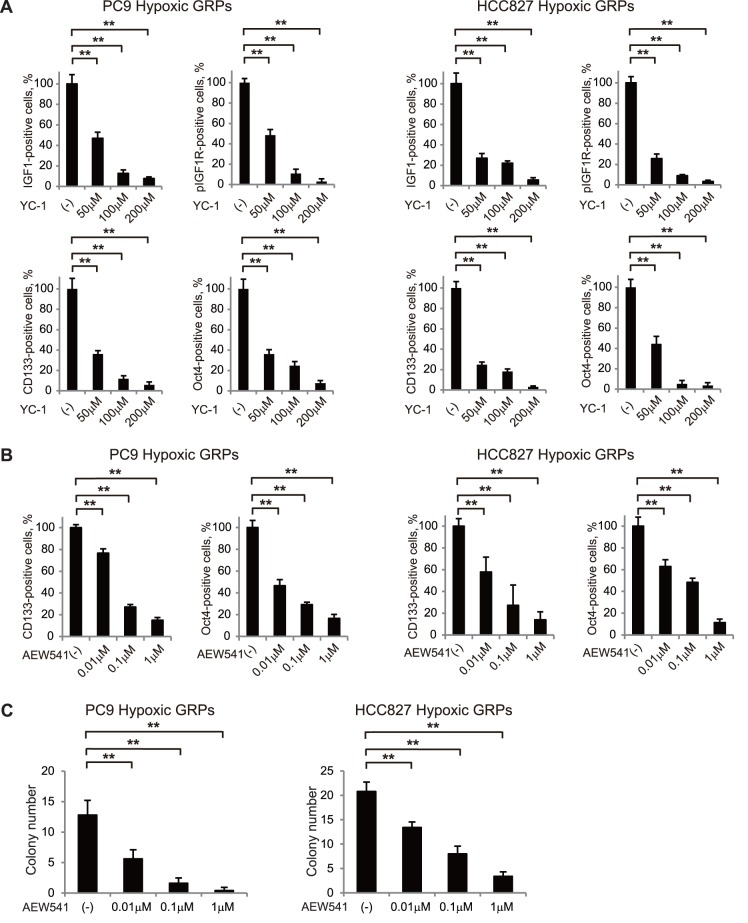
Hypoxia regulates IGF1 expression through HIF1α, and the inhibition of HIF1α or IGF1R decreased CD133- and Oct4-positive GRPs under hypoxia. **A.** HIF1α expression was suppressed in PC9 or HCC827 hypoxic GRPs by treatment with 50 µM, 100 µM, and 200 µM YC-1 in Lab-Tek chamber slides. Immunofluorescence staining for IGF1, phosphorylated IGF1R (pIGF1R), CD133, and Oct4 was then performed. The numbers of IGF1-, pIGF1R-, CD133-, and Oct4-positive cells were counted, and the ratio of these cells was calculated in five fields from each experiment. **p<0.001. **B.** PC9 or HCC827 cells were incubated with 1 or 2 µM gefitinib in the presence or absence of 0.01 µM, 0.1 µM, or 1 µM of the IGF1R inhibitor AEW541 under hypoxic conditions for 72 h in Lab-Tek chamber slides. Immunofluorescence staining for CD133 and Oct4 was then performed. The numbers of CD133- and Oct4-positive cells were counted, and the ratio was calculated in five fields for each experiment. **p<0.001. **C.** PC9 or HCC827 cells were plated in 10-cm plates and allowed to adhere for 24 h. Cells were then incubated with 1 or 2 µM gefitinib in the presence or absence of 0.01 µM, 0.1 µM, or 1 µM of AEW541 under hypoxic conditions for 18 or 11 days. The numbers of colonies were then counted.

To further investigate the effects of hypoxia on the upregulation of IGF1 and activation of IGF1R, we downregulated hypoxia-inducible factor 1α (HIF1α) expression in hypoxic GRPs by using the specific HIF1α inhibitor YC-1. Treatment with YC-1 inhibited HIF1α expression significantly in both PC9 and HCC827 cells under the hypoxia in a dose-dependent manner (Figure S4 in Information S1). As shown in Figure 6A, the inhibition of HIF1α by YC-1 treatment also suppressed IGF1 expression in hypoxic GRPs, and significantly reduced IGF1R phosphorylation on GRPs under the hypoxia. Furthermore, YC-1 treatment significantly decreased the population of CD133- and Oct4- positive hypoxic GRPs. Finally, treatment with the IGF1R inhibitor AEW541 significantly decreased the population of CD133- and Oct4-positive GRPs in a dose-dependent manner after gefitinib exposure under hypoxic conditions (Figure 6B). In addition, the number of colonies consisting of GRPs was suppressed significantly by AEW541 under hypoxic conditions (Figure 6C). Taken together, these findings strongly suggest that hypoxia regulates IGF1 expression through HIF1α, and that upregulated IGF1 induces the activation of IGF1R and increases the gefitinib-resistant CSCs in *EGFR* mutation-positive NSCLC cells under the hypoxia.

## Discussion

In this study, we exposed the NSCLC cell lines PC9 and HCC827 to 1 µM gefitinib under normoxic or hypoxic condition. Whereas most cells were killed within a few days of exposure to gefitinib, a small fraction of viable cells, referred to as GRPs, could survive 7 days later. These cells highly expressed stem cell genes, including CD133, Oct4, Sox2, Nanog, CXCR4, and ALDH1A1 and showed sphere-forming ability *in vitro* and high tumorigenic potential *in vivo*. IGF1 expression was upregulated in GRPs, and IGF1R was phosphorylated on GRPs. Importantly, hypoxia upregulated IGF1 expression, promoted the activation of IGF1R on GRPs, and significantly increased sphere-formation, tumorigenicity *in vivo*, and the population of CD133- and Oct4-positive GRPs. Furthermore, inhibition of HIF1α by YC-1 treatment significantly reduced IGF1 expression and phosphorylated IGF1R-expressing GRPs. Finally, treatment with YC-1 or the IGF1R inhibitor AEW541 significantly decreased the population of CD133- and Oct4-positive GRPs after gefitinib exposure under hypoxic conditions. Taken together, our findings suggest that GRPs are enriched for gefitinib-resistant stem cells, and hypoxia is involved in the persistence of the gefitinib-resistant stem cell population in *EGFR* mutation-positive NSCLCs through upregulation of IGF1 and the activation of the IGF1R pathway.

CD133 is considered an important marker of CSCs in NSCLC [5], and Oct4 expression has been shown to maintain CSC properties in lung cancer-derived CD133-positive cells [3]. Sox2 and Nanog are also known to be major stem cell pluripotency regulators [24]. Several recent reports implicate CSCs in the resistance to gefitinib in NSCLC [25], [26]. It has been demonstrated that gefitinib-resistant lung cancer cells exhibited strong sphere-forming activity and high CXCR4 expression, and isolated CXCR4-positive cells were responsible for the features of CSCs [26]. Aldehyde dehydrogenase 1 (ALDH1A1) has also been associated with the CSC phenotype [25]. These evidences are consistent with our current findings that GRPs after gefitinib exposure were highly enriched for stem cell genes, such as CD133, Oct4, Sox2, Nanog, CXCR4, and ALDH1A1. In our cell model, GRPs enriched for CD133 and Oct4 could survive under a high concentration of gefitinib, resumed proliferating, and eventually showed normal proliferation. Thus, GRPs can cause tumor relapse and regeneration during treatment with gefitinib. Targeting GRPs with stem cell properties is required for preventing the recurrence of NSCLCs treated with EGFR-TKIs.

Most of the previously reported NSCLC cells that were resistant to EGFR-TKI were established using the stepwise escalation of EGFR-TKI concentrations [27], [28]. Specifically, gefitinib concentrations were increased stepwise from 1 to 100 nM when cells resumed growth kinetics that were similar to untreated parental cells [27]. Acquired resistance by genetic alterations, such as the *EGFR* T790M mutation or amplified *MET*, were caused by the stepwise escalation method of EGFR-TKI exposure after prolonged treatment [27], [28]. However, resistant cells with stem cell properties were not observed with the stepwise escalation method. It was reported previously that the population of CD133-positive lung CSCs decreased during differentiation, and that differentiated cells lost stem cell properties such as self-renewal and tumorigenic potential [5]. Furthermore, recent studies suggest that CSCs are maintained in a quiescent state, and that quiescence is a critical factor for the resistance of CSCs to chemotherapy and targeted therapies [29]. Therefore, it is not possible to find stem-like features in CSCs after resistant cells, created using the stepwise escalation of EGFR-TKI concentrations, resumed proliferation comparable to untreated parental cells, since differentiated cells might have lost the stem cell-like properties. Sharma et al. recently treated PC9 cells with high concentration of erlotinib (2 µM) [2]. They reported that, whereas most cells were killed within a few days of exposure to erlotinib, a small number of viable cells could be detected 9 days later. The remaining cells were extremely quiescent, and expressed the CSC marker CD133. These reports are consistent with our current findings that GRPs were quiescent and highly enriched for stem cell genes after exposure to high concentrations of gefitinib for 7 days, and exhibited an increased potential for tumorigenicity *in vivo*. Shien et al. also reported recently that clonal gefitinib-resistant HCC827 cells after exposure to high concentration of gefitinib (2 µM) exhibited stem cell properties and ALDH1A1 overexpression [30]. This type of resistant cell with stem cell features was observed only after exposure to high concentrations of gefitinib, whereas the *EGFR* T790M mutation or *MET* amplification were found after the stepwise escalation method [30]. These findings are also consistent with our current results and previous reports.

In our study, hypoxia, known to be an important factor in stem cell niches, increased the population of stem cell marker-positive PC9 and HCC827 GRPs, which showed gefitinib resistance and markedly activated IGF1R. Activation of IGF1R induced by hypoxia might be mediated by HIF1α, which is a key transcription factor in the hypoxic response, since upregulation of the phosphorylated IGF1R-expressing GRPs was significantly suppressed by the downregulation of HIF1α expression using the specific inhibitor YC-1. Our findings also demonstrated that inhibition of IGF1R signaling reduced the population of gefitinib-resistant CSCs under hypoxic conditions. These findings indicate that hypoxia plays a key role in the persistence and drug resistance of the CSC phenotype by regulating IGF1R activation under gefitinib treatment in NSCLC. Eliasz et al. reported that hypoxia upregulated IGF1 and Notch1 expression in NSCLC cells, and that HIF1α-induced Notch1 promoted the survival of NSCLC cells under hypoxic conditions by activating the IGF1R pathway [31]. Notch signaling promotes the self-renewal of CSCs in several malignancies [32]. These studies are also consistent with our current findings, although Notch1 expression was not examined in our study. Targeting IGF1R may be a promising strategy for effectively controlling gefitinib-resistant CSCs in the hypoxic microenvironment and overcome hypoxia-induced resistance to EGFR-TKIs in NSCLC with an activating *EGFR* mutation.

Recently, IGF1R has been recognized as a drug target for NSCLC [18]. A clinical phase II trial was conducted to examine anti-IGF1R antibody CP-751,871 in combination with standard first-line chemotherapy comprising carboplatin and paclitaxel in advanced NSCLC [21], [33]. Furthermore, a randomized phase II trial of the combination of erlotinib, EGFR-TKI, with placebo or an R1507, another anti-IGF1R monoclonal antibody, was also performed for advanced NSCLC [34]. Although a multicenter phase III trial was discontinued because of the increased risk for severe toxicities of IGF1R antibody [35], phase II trials suggested that high free serum IGF1 levels are correlated with better prognosis in NSCLC patients undergoing combined therapy with erlotinib and IGF1R inhibitor [36]. Consistent with these clinical findings, IGF1 expression was markedly upregulated in both PC9 and HCC827 hypoxic GRPs in our cell model, and treatment with IGF1R inhibitor was effective against gefitinib-resistant cells. Although additional studies are necessary, circulating serum IGF1 level may be a predictive biomarker that can be used to identify patients who could benefit from treatment with IGF1R inhibitor.

To the best of our knowledge, this is the first study to reveal that hypoxia contributes to gefitinib resistance by regulating the CSC population mediated by IGF1R signaling activation in NSCLC with an activating *EGFR* mutation. We suggest that a combination of EGFR-TKI with IGF1R inhibitor or hypoxia-targeting approach would be more effective than either one alone. Furthermore, our results provide an insight into the mechanisms underlying intrinsic resistance to EGFR-TKIs in NSCLC with the activating *EGFR* mutation. However, further studies are required to verify our results.

## Supporting Information

Information S1 **Supplementary Materials and Methods; Figure S1. Analysis of microsatellites.** The GenomeLab Human STR Primer set contains 12 short tandem repeat (STR) primer pairs that amplify specific loci in the human genome (eleven STR loci, plus Amelogenin). The amplified loci are D3S1358, D7S820, D8S1179, D13S317, D16S539, D18S51, CSF1PO, Penta E, TPOX, Penta D, TH01, and Amelogenin. Analyses of the genomic DNA from parental cells and GRPs of PC9 and HCC827 cells are shown; **Figure S2. Immunofluorescence staining for phospho-Src, EGFR, and HER3.** PC9 or HCC827 cells, growing on Lab-Tek chamber slides with or without 1 or 2 µM gefitinib for 72 h, were fixed and then incubated with primary antibodies against phospho-Src, pEGFR, and pHER3, followed by incubation with secondary antibodies labeled with Alexa Fluor 488 goat anti-rabbit IgG (green). Cell nuclei were stained with DAPI (blue). Images were captured using an Axioplan 2 imaging system with AxioVision software. Images used to compare parental cells and GRPs were acquired using the same instrument settings and exposure times, and were processed similarly; **Figure S3. Knockdown of IGF1 expression by siRNA.** IGF1 expression was knocked down in PC9 or HCC827 hypoxic GRPs by using small interfering RNA (siRNA), and quantitative RT-PCR was performed with primers specific for IGF1 in PC9 or HCC827 parental cells and hypoxic GRPs. Two different specific siRNAs and one non-specific control were used. Data were normalized to actin expression. **p<0.001, *p<0.01, # p<0.05; **Figure S4. Inhibitory effect of YC-1 on HIF1α expression.** PC9 or HCC827 cells were grown on Lab-Tek chamber slides with or without 50 µM, 100 µM, and 200 µM YC-1 (HIF1α inhibitor) under hypoxic conditions for 18 h, and fixed. They were then incubated with primary antibodies against HIF1α, followed by Alexa Fluor 594-labeled goat anti-mouse IgG secondary antibody (red). Cell nuclei were stained with DAPI (blue). Images were captured using an Axioplan 2 imaging system with AxioVision software. All images were acquired using the same instrument settings and exposure times, and were processed similarly. The numbers of HIF1α-positive cells were counted, and the ratio of these cells was calculated in five fields for each experiment. Treatment with YC-1 significantly decreased the number of HIF1α-positive cells in a dose-dependent manner. **p<0.001.
(DOCX)
