# Supplementary material for: Hypoxia Increases Gefitinib-Resistant Lung Cancer Stem Cells through the Activation of Insulin-Like Growth Factor 1 Receptor
Source: PLoS One. 2014 Jan 28;9(1):e86459. doi: 10.1371/journal.pone.0086459 (PMC3904884; doi:10.1371/journal.pone.0086459)
Supplement: Information S1 — Supplementary Materials and Methods; Figure S1. Analysis of microsatellites. The GenomeLab Human STR Primer set contains 12 short tandem repeat (STR) primer pairs that amplify specific loci in the human genome (eleven STR loci, plus Amelogenin). The amplified loci are D3S1358, D7S820, D8S1179, D13S317, D16S539, D18S51, CSF1PO, Penta E, TPOX, Penta D, TH01, and Amelogenin. Analyses of the genomic DNA from parental cells and GRPs of PC9 and HCC827 cells are shown; Figure S2. Immunofluorescence staining for phospho-Src, EGFR, and HER3. PC9 or HCC827 cells, growing on Lab-Tek chamber slides with or without 1 or 2 µM gefitinib for 72 h, were fixed and then incubated with primary antibodies against phospho-Src, pEGFR, and pHER3, followed by incubation with secondary antibodies labeled with Alexa Fluor 488 goat anti-rabbit IgG (green). Cell nuclei were stained with DAPI (blue). Images were captured using an Axioplan 2 imaging system with AxioVision software. Images used to compare parental cells and GRPs were acquired using the same instrument settings and exposure times, and were processed similarly; Figure S3. Knockdown of IGF1 expression by siRNA. IGF1 expression was knocked down in PC9 or HCC827 hypoxic GRPs by using small interfering RNA (siRNA), and quantitative RT-PCR was performed with primers specific for IGF1 in PC9 or HCC827 parental cells and hypoxic GRPs. Two different specific siRNAs and one non-specific control were used. Data were normalized to actin expression. **p<0.001, *p<0.01, # p<0.05; Figure S4. Inhibitory effect of YC-1 on HIF1α expression. PC9 or HCC827 cells were grown on Lab-Tek chamber slides with or without 50 µM, 100 µM, and 200 µM YC-1 (HIF1α inhibitor) under hypoxic conditions for 18 h, and fixed. They were then incubated with primary antibodies against HIF1α, followed by Alexa Fluor 594-labeled goat anti-mouse IgG secondary antibody (red). Cell nuclei were stained with DAPI (blue). Images were captured using an Axioplan 2 imaging system wit [file pone.0086459.s001.docx]

**Hypoxia Increases Gefitinib-Resistant Lung Cancer Stem Cells through the Activation of Insulin-like Growth Factor 1 Receptor**

**Akiko Murakami, Fumiyuki Takahashi, Fariz Nurwidya, Isao Kobayashi, Kunihiko Minakata, Muneaki Hashimoto, Takeshi Nara, Motoyasu Kato, Ken Tajima, Naoko Shimada, Shin-ichiro Iwakami, Mariko Moriyama, Hiroyuki Moriyama, Fumiaki Koizumi, and Kazuhisa Takahashi**

**Supporting information**

**Supplementary Materials and Methods**

**Analysis of microsatellites**

The genetic identity of GRPs and parental cells was confirmed using the GenomeLab Human STR Primer set from Beckman Coulter (Fullerton, CA, USA) according to the manufacturer’s instructions.

**Immunofluorescence**

Phospho-Src (Tyr416), phospho-EGFR (Try1068), and phospho-HER3 (Y1289) antibodies were purchased from Merck Millipore (Billerica, MA, USA), Cell Signaling (Danvers, MA, USA), and Abcam (Cambridge, UK), respectively.

**Supplementary Figure Legends**

**Figure S1.**

**Analysis of microsatellites**

The GenomeLab Human STR Primer set contains 12 short tandem repeat (STR) primer pairs that amplify specific loci in the human genome (eleven STR loci, plus Amelogenin). The amplified loci are D3S1358, D7S820, D8S1179, D13S317, D16S539, D18S51, CSF1PO, Penta E, TPOX, Penta D, TH01, and Amelogenin. Analyses of the genomic DNA from parental cells and GRPs of PC9 and HCC827 cells are shown.

**Figure S2.**

**Immunofluorescence staining for phospho-Src, EGFR, and HER3**

PC9 or HCC827 cells, growing on Lab-Tek chamber slides with or without 1 or 2 μM gefitinib for 72 h, were fixed and then incubated with primary antibodies against phospho-Src, pEGFR, and pHER3, followed by incubation with secondary antibodies labeled with Alexa Fluor 488 goat anti-rabbit IgG (green). Cell nuclei were stained with DAPI (blue). Images were captured using an Axioplan 2 imaging system with AxioVision software. Images used to compare parental cells and GRPs were acquired using the same instrument settings and exposure times, and were processed similarly.

**Figure S3.**

**Knockdown of IGF1 expression by siRNA**

IGF1 expression was knocked down in PC9 or HCC827 hypoxic GRPs by using small interfering RNA (siRNA), and quantitative RT-PCR was performed with primers specific for IGF1 in PC9 or HCC827 parental cells and hypoxic GRPs. Two different specific siRNAs and one non-specific control were used. Data were normalized to actin expression. ** p < 0.001, * p < 0.01, # p < 0.05.

**Figure S4.**

**Inhibitory effect of YC-1 on HIF1α expression**

PC9 or HCC827 cells were grown on Lab-Tek chamber slides with or without 50 μM, 100 μM, and 200 μM YC-1 (HIF1α inhibitor) under hypoxic conditions for 18 h, and fixed. They were then incubated with primary antibodies against HIF1α, followed by Alexa Fluor 594-labeled goat anti-mouse IgG secondary antibody (red). Cell nuclei were stained with DAPI (blue). Images were captured using an Axioplan 2 imaging system with AxioVision software. All images were acquired using the same instrument settings and exposure times, and were processed similarly. The numbers of HIF1α-positive cells were counted, and the ratio of these cells was calculated in five fields for each experiment. Treatment with YC-1 significantly decreased the number of HIF1α-positive cells in a dose-dependent manner. ** p < 0.001.

**Figure S1.**


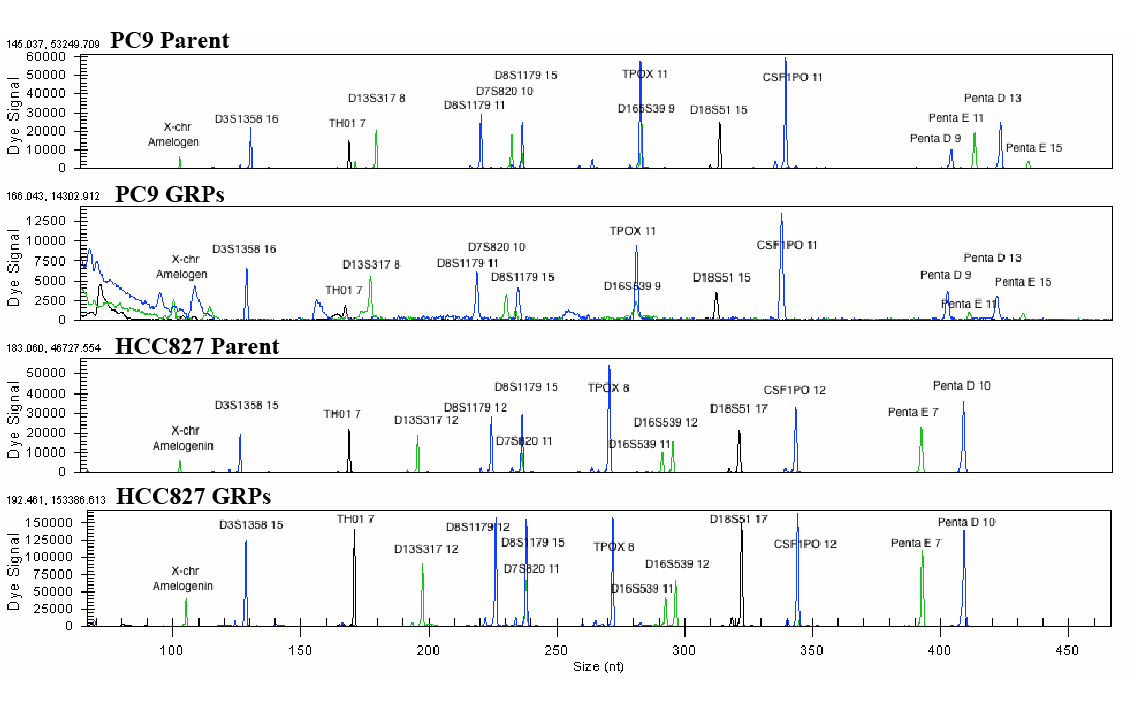


**Figure S1. tif**

**Figure S2.**

**
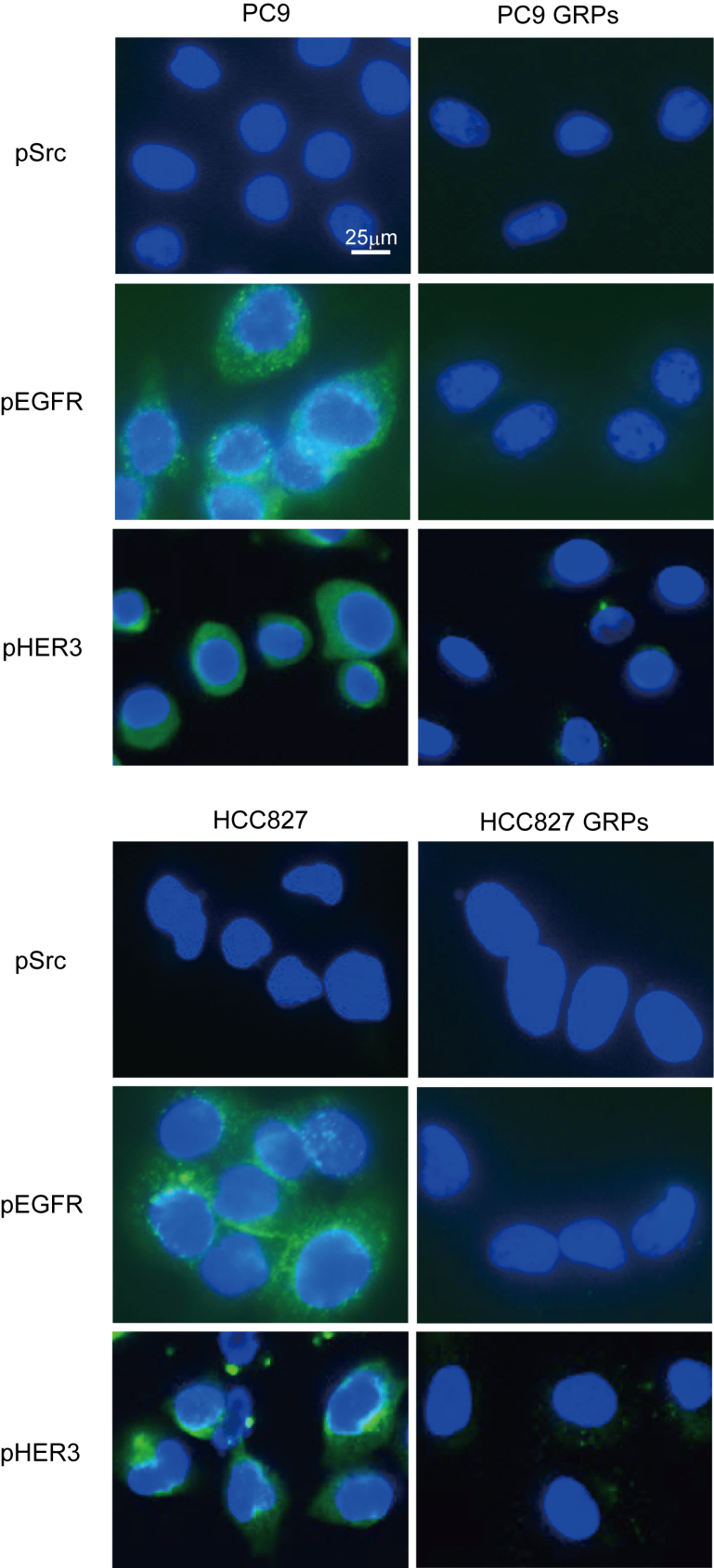
**

**Figure S2. tif**

**Figure S3.**

**
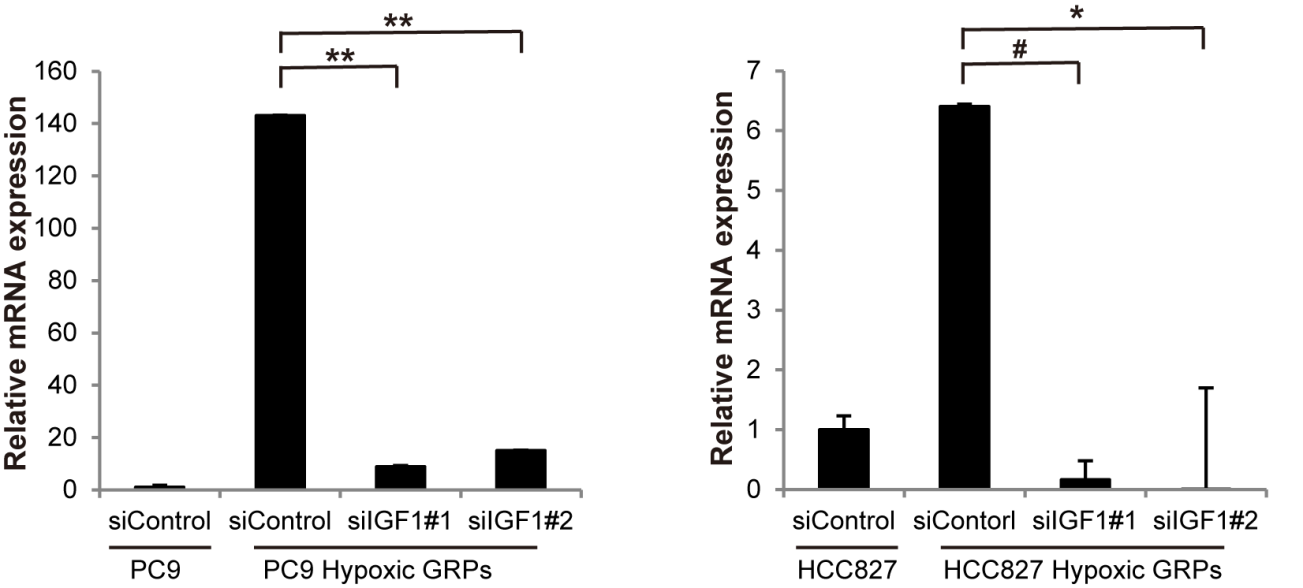
**

**Figure S3. tif**

**Figure S4.**


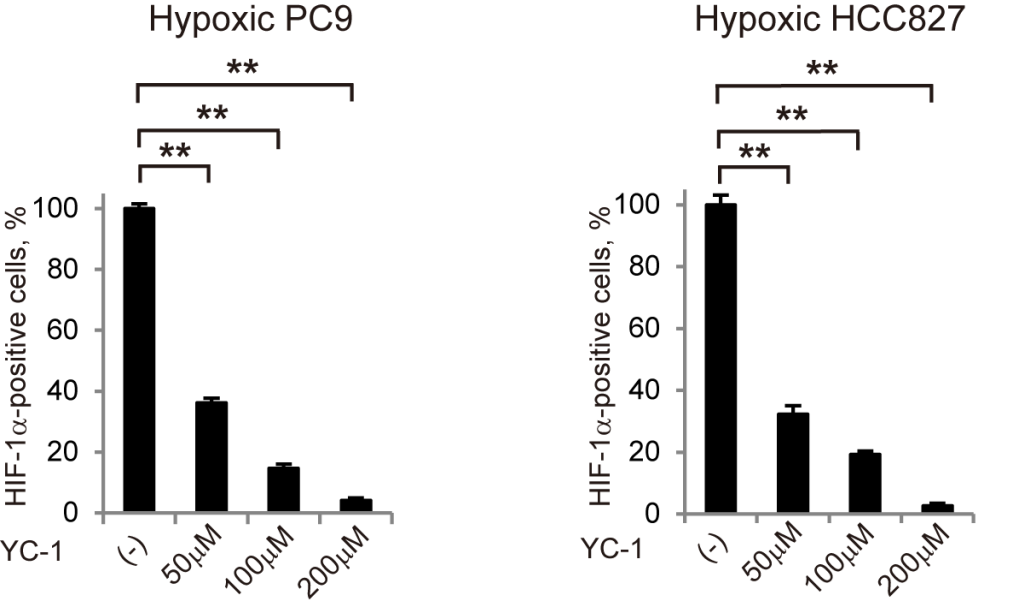


**Figure S4. tif**
